# Spatial genetic features of eastern oysters (*Crassostrea virginica* Gmelin) in the Gulf of Mexico: northward movement of a secondary contact zone

**DOI:** 10.1002/ece3.1064

**Published:** 2014-04-10

**Authors:** Joel D Anderson, William J Karel, Christopher E Mace, Brian L Bartram, Matthew P Hare

**Affiliations:** 1 Perry R. Bass Marine Fisheries Research Station, Texas Parks and Wildlife3864 FM 3280, Palacios, Texas, 77465; 2 Rockport Marine Lab, Texas Parks and Wildlife824 S. Fuqua St., Rockport, TX, 78382; 3 Department of Natural Resources, Cornell University213 Bruckner Hall, Ithaca, NY, 14853

**Keywords:** *Crassostrea virginica*, Gulf of Mexico, hybridization, microsatellite, secondary contact, vicariance

## Abstract

The eastern oyster (*Crassostrea virginica* Gmelin) is an economically and ecologically valuable marine bivalve occurring in the Gulf of Mexico. This study builds upon previous research that identified two divergent populations of eastern oysters in the western Gulf of Mexico. Allelic and genotypic patterns from 11 microsatellite markers were used to assess genetic structure and migration between the previously described oyster populations in Texas. The main findings are as follows: (1) there are two distinct populations (*F*_ST_ = 0.392, *P* < 0.001) of oysters that overlap in the Corpus Christi/Aransas Bay estuarine complex in Texas, (2) the distribution of genotypes among individuals in the contact zone suggests limited hybridization between populations, (3) the variables of salinity, temperature, dissolved oxygen, turbidity and depth are not correlated with allele frequencies on reefs in the contact zone or when analyzed across Texas, and (4) there is little evidence of directional selection acting on the loci assayed here, although patterns at four markers suggested the influence of balancing selection based on outlier analyses. These results are consistent with long-term historical isolation between populations, followed by secondary contact. Recent hydrological changes in the area of secondary contact may be promoting migration in areas that were previously inhospitable to eastern oysters, and observed differences in the timing of spawning may limit hybridization between populations. Comparison of these findings with the results of an earlier study of oysters in Texas suggests that the secondary contact zone has shifted approximately 27 km north, in as little as a 23-year span.

## Introduction

**Figure 1:**
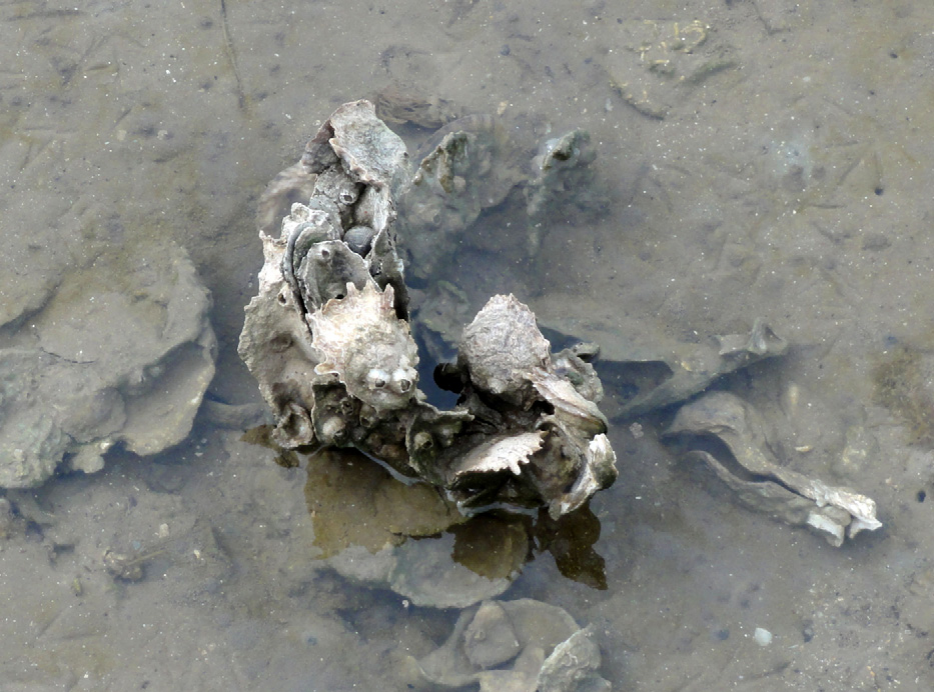
Eastern oysters are found in inshore habitats throughout the Gulf of Mexico and provide habitat, shoreline stabilization, water filtration, and other ecosystem services.

The eastern oyster (*Crassostrea virginica* Gmelin) is an economically and ecologically valuable organism common to estuarine habitats throughout the Gulf of Mexico and North American east coast. The overall ex-vessel value of eastern oysters in the United States was valued at over US$90 million in 2011 (NMFS 2012). In terms of ecosystem services, oysters provide nursery and foraging habitat for fishes and invertebrates (Peterson et al. 2003; Rodney and Paynter 2006), stabilize shorelines and provide erosion control (Meyer et al. 1997), improve water clarity and quality (Dame et al. 1984; Shpigel and Blaylock 1991; Porter et al. 2004; Newell et al. 2005), and function as primary consumers of suspended phytoplankton (Baird et al. 2004; Fig. 1). The economic and ecological value of oysters is unquestioned, yet eastern oysters have declined in some areas of their range to as little as 1% of their historical abundance based on estimates from commercial landings (Rothschild et al. 1994). While this magnitude of decline has not been recognized in the Gulf of Mexico, the decline in oysters in many Atlantic coast areas has resulted in a concentration of the industry in Gulf waters. The fishery supported by this organism in the Gulf of Mexico represented approximately 70% of the oyster fishery in the USA in 2011, versus 40% in 1981 (NMFS 2012). In addition to stresses caused by intensive harvest, oysters are subject to environmental and anthropomorphic pressures such as the presence of non-indigenous parasite diseases (Ewart and Ford 1993), sedimentation due to dredging operations (Rose 1973), water quality degradation (Lenihan and Peterson 1998), and loss of habitat (Rothschild et al. 1994; Lenihan and Peterson 1998). Moreover, near-shore estuarine habitats in which oysters reside are dynamic environments with dramatic fluctuations in water temperature, salinity, depth, dissolved oxygen and turbidity, all of which can influence mortality in eastern oysters. There is thus a paradoxical balance between the numerous ecosystem services provided by oysters, versus their ability to exist in extremely variable environmental conditions and under intensive harvest.

Given the dramatic environmental stresses experienced by oysters, the demographic connectivity and gene flow among adjacent reef systems is likely an important component of survival of oyster populations as a whole. Sessile marine invertebrates often have migratory life stages that limit localized genetic divergence, and allow for long distance gene flow. In the case of the eastern oyster, migration occurs during a 3-week planktonic larval stage prior to substrate settlement and transition to the sessile adult stage (Buroker 1983). Previous genetic examinations of population structure in the eastern oyster have been equivocal in regard to the number of populations occurring across the range of the species (Murray and Hare 2006). This ambiguity has been attributed to the highly stochastic nature of variation in genetic divergence created by neutral drift acting on multiple independent loci (Cunningham and Collins 1994; McDonald et al. 1996; Murray and Hare 2006), localized selection (Karl and Avise 1992), and secondary contact following a previously allopatric distribution (Murray and Hare 2006). Eastern oysters in the western Gulf of Mexico apparently comprise at least two distinct populations (Groue and Lester 1982; Buroker 1983; King et al. 1994; Hoover and Gaffney 2005). The transition between these populations occurs between the Laguna Madre of southern Texas, and estuaries further north (King et al. 1994; Varney et al. 2009), an area in which there are no obvious physical barriers to migration. King et al. (1994) speculated that the observed genetic divergence of eastern oyster populations between northern and southern estuaries in Texas was potentially the result of a historically small effective population size within the semi-isolated Laguna Madre ecosystem, resulting in genetic drift between this system and systems further north. However, hydrological changes in this area associated with the 1949 opening of the Gulf Intracoastal Waterway (GIWW) have increased the potential for gene flow to occur between the Laguna Madre and proximate oyster reefs to the north and imply the possibility of secondary contact between once isolated populations. The sharp clines in eastern oyster allele frequencies demonstrated by King et al. (1994) across a small spatial scale (26 km), if persistent, suggest the potential for contemporary secondary contact between divergent populations, with admixture limited by low hybrid fitness or limited opportunities for hybridization. The previous studies of eastern oysters in Texas were not explicitly designed to address the fine-scale patterns of distribution within the transition zone between populations, and did not test mechanistic hypotheses explaining genetic divergence. In the event of a sympatric or partially overlapping distribution, the degree of hybridization and introgression between populations could have implications for management. The successful transplant and aquaculture of oysters for the commercial oyster fishery, as well as conservation efforts to preserve viable oyster reef habitat (reef restoration) both will benefit from improved understanding of the mechanisms maintaining genetically discrete populations.

Hybrid-zone analysis has historically been used to assess the taxonomic status of divergent populations, for example by categorizing the observed admixture between populations as either unimodal or bimodal with respect to a genotypic hybrid index (Hewitt 1988; Jiggins and Mallet 2000). Unimodal hybrid zones are those in which recombinant genotypes are common and individuals mate randomly within the zone (Jiggins and Mallet 2000). Such cases are common in secondary contact zones where divergence time has been too short for reproductive barriers to develop between populations. The resulting population is composed of offspring that have unique hybrid genotypes, which become more common in the middle of the zone. Bimodal hybrid zones are those in which recombinant genotypes are relatively rare. Such cases imply the presence of reproductive barriers between populations and result in offspring which carry primarily genotypes which resemble one or the other parental population (Harrison and Bogdanowicz 1997). The two hybrid-zone patterns provide a test for alternative hypotheses on the realization of gene flow through a contact zone, and in the case of a bimodal zone, allow for predictions of whether incipient speciation may be occurring. In this study, genetic data were used to characterize the geographical distribution of divergent eastern oyster populations in Texas, as well as measure gene flow between them. A macrogeographic sampling scheme covering the extent of oysters in Texas was followed by microgeographic sampling targeted to the area of transition between populations. The objectives of this study were threefold: (1) describe broad- and fine-scale population structure of oyster populations in the western Gulf of Mexico, (2) test for gene flow and admixture in oyster populations near the genetic transition zone, and (3) explore environmental factors and historical biogeography which may influence the distribution of each population in the transition zone.

## Materials and Methods

### Sample collection and laboratory procedures

**Figure 2:**
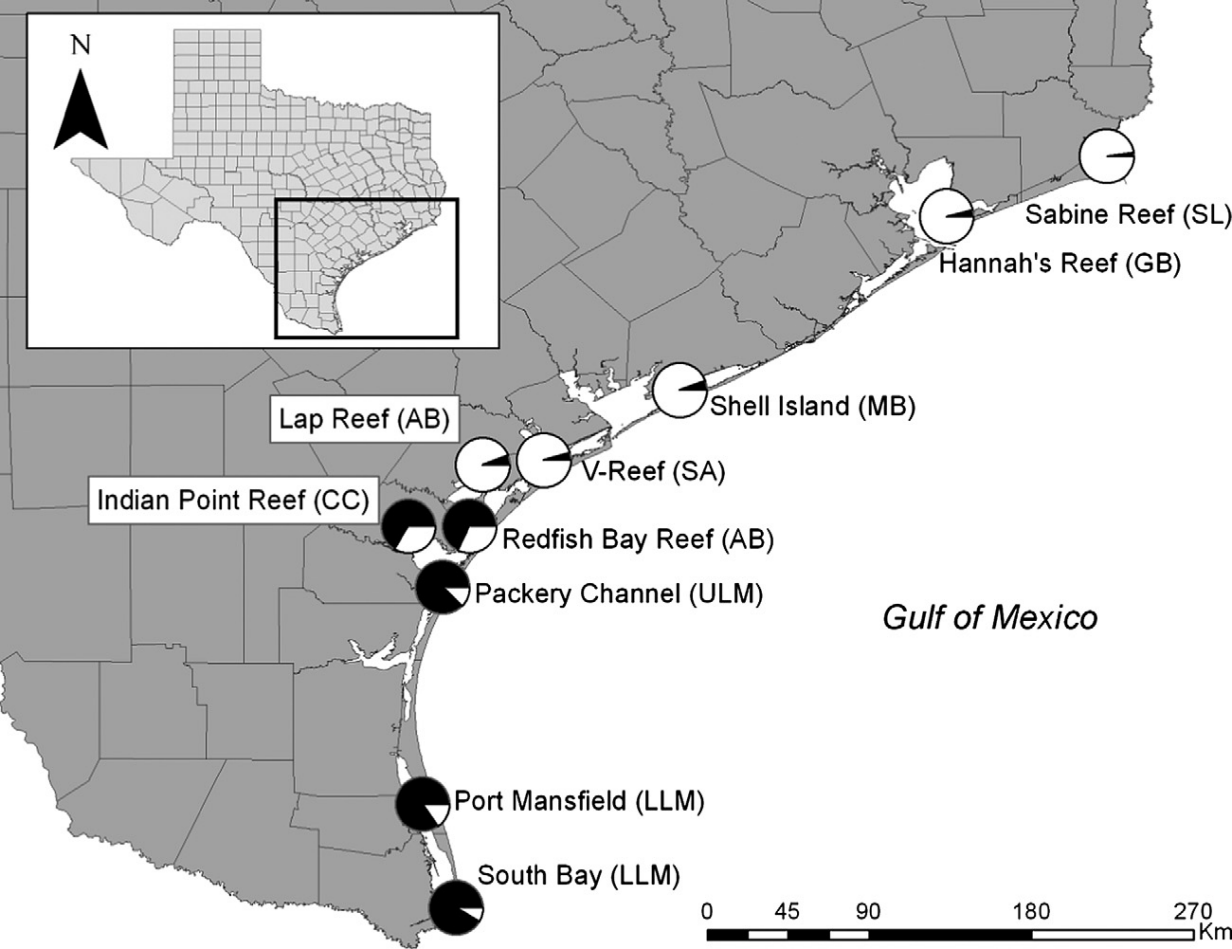
The structure cluster analysis of macrogeographic samples of eastern oysters (*Crassostrea virginica*). Samples taken on the northern coastline between Sabine Lake and San Antonio Bay had mean individual admixture scores (*Q*) comprised of *Q* > 70% for a single genetic cluster (white), while southern coastline samples in the upper and lower Laguna Madre were primarily comprised of an opposing genetic cluster (black). Samples in lower Aransas Bay (Redfish Bay Reef) and Corpus Christi Bay (Indian Point Reef) had less than *Q* < 70% relative to both genetic clusters.

Eastern oyster sample collection was broken up into two phases. The first phase consisted of sampling single oyster reefs residing in each major estuary along the coast of Texas, with the goal of identifying broad-scale geographic patterns of population structure (hereafter “macrogeographic” samples). For macrogeographic sampling, oyster specimens were obtained by bottom dredge in 10 sampling locales which covered the extent of the inland (littoral) zone in Texas, from Sabine Lake in the north to the Brownsville ship channel in the south (Fig. 2, Table 1). The targeted sample size for this phase was *n* = 45 oysters for each locale, and oysters were collected indiscriminately with respect to age. Samples for this phase were taken on various dates in the fall and spring of 2011/2012.

**Table 1:** Environmental variables for macrogeographic eastern oyster samples in the western Gulf of Mexico, the mean value of individual admixture scores (*Q*) for each sample using the 2-cluster solution from structure analysis, and the multilocus within-population component of genetic divergence (*F*_IS_, an asterisk indicates a significantly high value). The mean for each environmental variable was calculated from 30-year annual mean values (over all seasons)

Major Bay	Reef	Lat.	Long.	Temp (°C)	Salinity (‰)	*D*_*o*_ (mg/L)	Turbidity (NTU)	Cluster 1 (*Q*)	Cluster 2 (*Q*)	*F*_IS_
Sabine Lake	Sabine Reef	29.78	−93.92	23.3	11.8	7.8	25.8	0.97	0.03	0.02
Galveston	Hannah's Reef	29.48	−94.73	21.6	16.1	7.7	34.4	0.96	0.04	0.03
Matagorda	Shell Island	28.62	−96.05	23.6	18.9	7.1	58.0	0.94	0.06	0.11*
San Antonio	V-Reef	28.27	−96.76	22.2	22.8	7.6	18.6	0.95	0.05	0.13*
Aransas	Lap Reef	28.14	−97.04	22.6	18.8	7.3	16.5	0.93	0.07	0.11*
Corpus Christi	Redfish Bay	27.89	−97.11	25.2	27.4	8.1	14.4	0.32	0.68	0.13*
Corpus Christi	Indian Point Reef	27.86	−97.36	24.6	28.3	7.5	29.9	0.34	0.66	0.10*
Upper Laguna Madre	Packery Channel	27.64	−97.24	24.6	35	7.6	10.8	0.12	0.88	0.06
Lower Laguna Madre	Port Mansfield	26.55	−97.38	25.9	34	7.5	29.1	0.15	0.85	0.13*
Lower Laguna Madre	South Bay	26.02	−97.19	26.6	37.6	7.8	16.9	0.08	0.92	0.02

**Figure 3:**
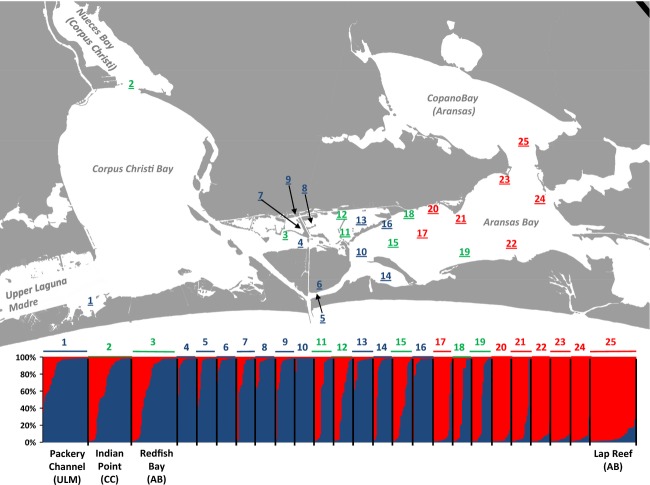
Structure bar plot results for 25 eastern oyster samples taken in Upper Laguna Madre, Corpus Christi Bay, and Aransas Bay, Texas. Structure analysis indicated two populations (*K*) in the sampled area, corresponding to blue and red bars that are sorted by *Q* within each sample. The numbers above each Structure group on the bar chart correspond to the sample locations indicated on the map. Samples which are labeled blue had primarily cluster 1 genotypes (cluster 1 *Q* > 0.7), samples in red had cluster 2 genotypes (cluster 2 *Q* > 0.7), and samples in green had significant mixtures of both populations (admixture *Q* < 0.7). Samples that are labeled with names below the bar chart are identical to similarly labeled samples in Figure 2.

Following a cursory analysis of genetic structure based on data collected during the macrogeographic sampling phase, the Corpus Christi/Aransas Bay area was identified as an area of contact between divergent populations. The second sampling phase had the goal of identifying fine-scale genetic structure in the area between Corpus Christi and Aransas bays (“microgeographic” samples). For this phase of sampling, 22 sample sites were chosen in the transition area using two criteria: (1) samples were chosen to give broad and representative coverage of the transition area, and (2) sample sites were in areas of known availability of oysters, such as large reefs or mid-bay structures that were likely to be inhabited by oysters (Fig. 3). Sampling for the microgeographic phase consisted of both bottom dredge and manual hand collection of *n* = 20 live oysters from each site. Microgeographic samples were taken on a single day in July 2012.

All oysters were immediately placed on ice after collection, for transport to Perry R. Bass Marine Fisheries Research Station (PRB) in Palacios, TX. Oysters were frozen for up to 2 weeks prior to tissue sample extraction. Tissue samples (1 cm^3^ of mantle tissue) were taken from each oyster and placed in 95% ethanol. Each specimen was then sub-sampled (approximately 20 mg tissue) for DNA extraction, and DNA was extracted with a Gentra Puregene Tissue Kit (Qiagen Inc., Valencia, CA). Elution volumes were based upon pellet size and averaged 200 *μ*L (larger pellets were dissolved in up to 300 *μ*L).

Primers from 11 microsatellite markers were used to amplify the corresponding variable repeat loci in each sample (Table 2). Each marker had one primer labeled with a WellRED dye (Sigma-Proligo, Boulder, CO) and amplified independently (nonmultiplexed) via polymerase chain reaction (PCR). Loci were amplified with a modified touchdown protocol and Ready-To-Go™ PCR beads (GE Healthcare, Piscataway, NJ) on a Techne Genius thermal cycler (Techne Inc., Princeton, NJ). Reactions consisted of 1 *μ*L template DNA (50 ng/*μ*L), 1 Ready-To-Go™ bead, and 24 *μ*L of forward and reverse primer cocktail (0.4 *μ*mol/L standard primer concentration of each primer), for a total of 25 *μ*L. The PCR protocol utilized for all reactions was as follows: an initial denature period of 2 min at 95°C, 10 cycles of primary amplification (95°C for 30 s, 55°C for 30 s lowering 1°C each cycle, and 72°C for 1 min), 20 cycles of secondary product amplification (95°C for 30 s, 50°C for 30 s, and 72°C for 1 min adding 3 s of extension per cycle), and a single final extension period of 7 min at 72°C. Amplified products were combined in groups with a 400-bp size standard and separated on a Beckman-Coulter CEQ8000® automated sequencer (Beckman Coulter Inc., Fullerton, CA). Fragment analysis was performed using Beckman-Coulter software, and all size calls were examined visually for accuracy.

**Table 2:** Locus statistics for microsatellite loci used to assay eastern oysters in this study. The number of alleles observed (alleles) was calculated over all samples, as were observed (*H*_*o*_) and expected (*H*_*e*_) heterozygosity, and *F*_IS_. The 95% confidence interval of *F*_IS_ was determined using a simulation/resampling procedure in fstat (Goudet 2001). The genotyping error rate (the frequency of duplicated sample runs which resulted in different allele size calls) was determined by comparison of samples run on multiple occasions

Locus	Alleles	*H*_*o*_	*H*_*e*_	*F*_IS_ (95% CI)	Genotyping error rate	Original reference
Cvi1a13	5	0.038	0.037	0.00 (na)	0.017	Reece et al. (2004)
Cvi1j14	10	0.321	0.284	0.00 (na)	0.000	Reece et al. (2004)
Cvi2i23	29	0.891	0.891	0.00 (na)	0.025	Reece et al. (2004)
Cvi2j24	25	0.727	0.847	0.14 (0.10–0.17)	0.042	Reece et al. (2004)
Cvi2k14	9	0.446	0.468	0.04 (0.00–0.08)	0.033	Reece et al. (2004)
Cvi2m4	11	0.164	0.186	0.11 (0.08–0.15)	0.008	Reece et al. (2004)
Cvi9	24	0.778	0.913	0.15 (0.11–0.18)	0.025	Brown et al. (2000)
Rucv52	6	0.377	0.427	0.12 (0.08–0.16)	0.042	Wang and Guo (2007)
Rucv53	6	0.303	0.316	0.04 (0.01–0.06)	0.008	Wang and Guo (2007)
Rucv74	9	0.568	0.649	0.12 (0.08–0.17)	0.000	Wang and Guo (2007)
Rucv89	22	0.525	0.587	0.10 (0.06–0.14)	0.017	Wang and Guo (2007)

### Microchecker analysis and locus statistics

Genotyping errors due to irregularities in marker amplification were assessed using the software microchecker (van Oosterhout et al. 2004). This analysis was conducted for each marker in each of the ten macro-geographic samples. Heterozygote deficiencies at microsatellite loci (Naciri et al. 1995; Huvet et al. 2000; Launey et al. 2002; Hedgecock et al. 2004; Astanei et al. 2005; Carlsson and Reece 2007) and null allele presence (Gaffney 1990; McGoldrick et al. 2000; Reece et al. 2004; Galindo-Sanchez et al. 2008) are common features of marine bivalve populations, and the genus *Crassostrea* in particular (reviewed in Galindo-Sanchez et al. 2008). The eleven loci chosen for genetic structure analysis satisfied the following criteria: (1) each marker had a mean estimated null allele frequency (average of all samples) <0.05, and (2) each marker showed evidence for genotyping errors due to allele stutter in one or fewer samples. Additionally, duplicate samples from 60 individuals from the macrogeographic survey were repeated (same DNA samples, run on two different occasions) to estimate the genotyping error rate for each marker locus, which was below 5% in each case.

The observed and expected heterozygosity, total number of alleles, and accordance with Hardy–Weinberg equilibrium (HWE) expectations within macrogeographic samples and among all loci were examined using fstat version 2.9.3 (Goudet 2001). The statistic *F*_IS_ is used here to describe the magnitude of any deviation from HWE. The confidence intervals of *F*_IS_ estimates were tested at each locus by jackknifing over all samples. Lack of statistical significance was assumed in cases where the confidence interval of *F*_IS_ overlapped 0.

### Genetic structure

The spatial genetic structure of oysters was evaluated with a combination approach using Bayesian cluster analysis (structure version 2.3.4, Pritchard et al. 2000) and traditional analysis of molecular variance (AMOVA, Excoffier et al. 1992). An initial structure run was performed using the admixture model with correlated allele frequencies (Falush et al. 2003), with both macro- and microgeographic samples included in order to determine the number of populations present in the overall sample. Comparative runs with the number of populations (*K*) >2 resulted in complex admixture patterns within all individuals, and lowered the resolution of admixture between the two main populations. Therefore, the number of populations in subsequent structure runs was constrained to *K* = 2. The two-population model is supported by five previous genetic analyses of oysters in Texas (Groue and Lester 1982; Buroker 1983; King et al. 1994; Hoover and Gaffney 2005; Varney et al. 2009). Admixture coefficient stability among individuals during the course the structure run was used to evaluate model convergence. Exploratory runs indicated that a burnin of 50,000 iterations followed by 450,000 sampling iterations was adequate to achieve convergence.

Following the initial exploratory analysis with structure, the presence of hierarchical genetic structure within the two-population framework was evaluated by AMOVA. Samples were grouped into “northern” and “southern” regions, based upon having sample mean admixture proportions (*Q*) favoring structure cluster 1 or 2, respectively. The initial AMOVA model included two hierarchical levels, that between the two regions (northern vs. southern) inferred using structure analysis, and secondarily among samples within regions. The significance of variance components in the AMOVA model were evaluated by 1000 permutations of the data, and variance components were used to estimate between region (*F*_*ct*_), and among-sample (*F*_*sc*_) genetic divergence. A post hoc examination of pairwise comparisons suggested that the most heavily admixed samples from the structure analysis were driving a significant value of *F*_*sc*_. Thus, these samples were removed from the AMOVA model iteratively (starting with the most heavily admixed sample), until the among-sample component of divergence was no longer significant. In this way, each sample was classified in one of three categories: northern (structure cluster 1), southern (structure cluster 2), or mixed. The samples which fell into the mixed group had individual mean admixture coefficients (*Q*) < 70% in relation to either structure cluster (neither cluster made up more than 70% of the sample). The unmixed samples had individual mean admixture of *Q* > 80% in each case. Variance partitioning and significance testing of the AMOVA model were carried out using ARLEQUIN v3.5 (Excoffier and Lischer 2010). After the iterative AMOVA procedure, a final structure analysis was carried out using the unmixed flanking samples as reference populations in the structure model, and allele frequencies from these samples were used to improve ancestry estimation in mixed samples.

The existence of linkage disequilibrium (LD) was tested using the likelihood-ratio test of Excoffier and Slatkin (1998). The LD test was used to independently evaluate LD in both unmixed populations and in the mixed samples grouped together. Deviation from HWE was also tested in these groups using the method of Guo and Thompson (1992). The LD and HWE test statistics were corrected for tablewide significance using a sequential Bonferroni procedure.

The hybrid-zone analysis of Nielsen et al. (2003) was used to determine whether oysters in mixed samples demonstrated either a unimodal (hybridizing) or bimodal (mechanically mixed) distribution. First, the distribution of admixture categories among the mixed samples were compared using a Kruskal–Wallis test for multiple samples (*k* = 7). Upon finding that the mixed samples had significantly similar distributions (see Results), the mixed samples were combined, and the expected distributions of individual admixture scores were generated from empirical data under both the hybrid model and the mechanical mixing model using the software hybridlab (Nielsen et al. 2006). The mechanical mixing model was simulated by generating random genotypes based on parental allele frequencies in the two unmixed groups (northern and southern) flanking the hybrid zone. The expected ratio of northern and southern genotypes in mixed samples were estimated from sample-specific mean *Q* values from structure runs. The hybrid model was simulated by generating random genotypes based upon observed allele frequencies within the mixed samples. The simulated data under the alternative models were used in a structure analysis with the number of populations constrained to *K* = 2. Individual admixture scores from each simulation were used to generate distribution functions for simulated data, and these distributions were compared with observed admixture data using the Kolmogorov–Smirnov nonparametric test, as implemented in sas 9.2 (SAS Inst., Inc., Cary, NC). Additionally, individual admixture scores from structure analysis of mixed samples were used to construct a composite hybrid index plot. Each individual from admixed samples was classified as existing in one of 10 hybrid index categories (0.1–1.0) based on structure*Q*, with a score of 0.5 indicating an F1 hybrid. The shape of the hybrid index plot was then qualitatively examined for evidence of either a unimodal or bimodal hybrid zone (Jiggins and Mallet 2000).

### Detection of loci under selection

An alternative to the hypothesis of secondary contact to explain genetic divergence between overlapping populations is that divergence evolved in place and that divergent loci are linked to adaptive loci responding to variability in environmental cues. The adaptive divergence hypothesis (*H*_*a*_) was tested a number of ways. First, the model-based outlier test of Beaumont and Nichols (1996) was implemented using the software fdist2 (available online at: http://www.maths.bris.ac.uk/∼mamab/, last accessed 08/16/13). The genomic mean *F*_ST_ was estimated as in Beaumont and Nichols (1996), and this value was used to anchor simulations of the variance in *F*_ST_ as a function of heterozygosity. Simulated data were then used to construct confidence intervals around the mean value of *F*_ST_ at increasing levels of heterozygosity, and the observed data points (loci) were plotted relative to the expected distribution of *F*_ST_. Simulated output and the observed data were plotted in R (R Development Core Team 2011).

The cline analysis method of Strand et al. (2012) was used to identify significant differences in allele frequency cline location (coincidence) and slope (concordance) across loci. In contrast to the previous outlier tests, the method described by Strand et al. (2012) is not model-based, rather loci under selection are detected by observed deviation from the distribution of mean clinal coincidence/concordance (Co-Co plot, Strand et al. 2012). The criteria of Strand et al. (2012) were used to identify clinal loci as those in which: (1) a significant pattern of isolation by distance could be demonstrated, and (2) a broken-stick regression model (piecewise regression) was a significant improvement over linear regression in the model fit of latitude versus allele frequency. We used the allele frequency data presented in Table 2 of King et al. (1994) to include clinal analysis of previously published allozyme markers at similar sampling locations. For clinal loci, the initial parameters for the piecewise regression model (allele frequency north and south of the cline, and geographic boundaries of the cline based on latitude) were estimated visually from allele frequency plots across samples, and then improved using the non-linear logistic (nlin) procedure in sas 9.2. The slope on the north and south sides of the allele frequency cline were constrained to 0 in the “twice-broken-stick” model (based upon the assumption that allele frequencies in unmixed northern and southern samples were consistent across samples). Exact values for the midpoint latitude and slope of the cline were calculated using the improved parameters from the piecewise regression. Cline slopes were log-transformed as described by Strand et al. (2012), and confidence curves were generated around all Co-Co data points using 2-dimensional kernel density curves. Bandwidths for the density curves were estimated using the biased cross-validation bandwidth matrix selector (Sain et al. 1994) as implemented in the R package ks (Duong 2007). Univariate differences in cline midpoints and slopes between microsatellite and allozyme data were tested using t-tests assuming unequal variance between classes in SAS. There were differences in exact sampling locations between the previous allozyme study (King et al. 1994) and the locations sampled here. Therefore, a second Co-Co data analysis was conducted using only those sample locations that were identical between studies in order to account for potential differences in cline concordance/coincidence caused by sampling different sites.

An additional approach to testing the adaptive divergence *H*_*a*_ was to assess the impact of environmental variables on both macrogeographic and microgeographic oyster population structure. The hierarchical Bayesian model of Foll and Gaggiotti (2006) estimates sample-specific values of *F*_ST_ (as in Balding and Nichols 1995) but uses spatial variation in environmental parameters to construct model priors. Thus, suites of various environmental variables can be tested against one another, and each model can be evaluated via the posterior probability distribution. Environmental variables were derived from year-round sampling conducted by the Texas Parks and Wildlife Department Coastal Fisheries Division (TPWD), which includes randomized sampling of finfish and shellfish in 1′ latitude by 1′ longitude grids covering the extent of inshore waterways along the Texas coast. Organismal sampling is always coupled with standardized measures of water characteristics, using YSI water sampling probes (YSI, Inc., Yellow Springs, OH). Data from the years 1980 to 2012 were acquired for each grid, and long-term values of each water quality variable were calculated as the mean of all observations. Each grid was sampled an average of 65 times over the 32-year period. Environmental variables included in the macrogeographic analysis were water surface mean annual temperature (°C), mean summer water temperature (May – Sept, °C), mean annual salinity (‰), mean summer salinity (May – Sept, ‰), dissolved oxygen (mg/L), and turbidity (NTU; Table 2). Latitude was also included as a variable and was measured at the mid-point latitude for each sampled reef in the macrogeographic samples. Similar variables were used to examine microgeographic samples, with the added variable reef depth (Table 3). The software GESTE (available from http://www-leca.ujf-grenoble.fr/logiciels.htm, last accessed 02/06/2013) was used to run 2,000,000 sampling iterations following a 50,000 iteration burn-in. Proposal distributions were generated simultaneously during the burn-in period, using 10 pilot runs of 5000 iterations. Each possible multivariate combination of variables was considered, resulting in 64 potential models of environmental correlation in the macrogeographic analysis and 256 potential models in the microgeographic analysis.

**Table 3:** Environmental variables included in the microgeographic analysis of population structure (GESTE), and the mean structure*Q*-score of individuals sampled on 21 reefs in Aransas Bay, Texas. All environmental variables are measured in the field to the tenth (0.0) except turbidity, which is rounded to whole numbers. The reef grid number is the same as designated in Fig. 3

Reef grid	Temp (^o^C)	Summer temp	Salinity (‰)	Summer salinity	Dissolved oxygen (mg/L)	Turbidity (NTU)	Latitude	Depth (m)	Cluster 1 (*Q*)	Cluster 2 (*Q*)
4	23.6	29.2	28.6	28.4	7.9	17	27.9	0.5	0.05	0.95
5	22.4	28.1	28.5	28.5	7.9	41	27.9	3.4	0.10	0.91
6	24.8	29.8	28.0	28.0	8.8	16	27.9	0.9	0.04	0.96
7	22.1	29.9	27.7	27.7	8.9	21	27.9	0.5	0.19	0.81
8	26.4	28.7	25.8	25.8	7.4	18	27.9	0.7	0.07	0.93
9	24.1	28.8	25.4	25.4	8.6	14	27.9	0.9	0.13	0.87
10	23.6	28.7	27.1	27.3	7.7	12	27.9	1.9	0.02	0.98
11	24.8	30.0	23.5	23.5	9.0	14	27.9	0.6	0.35	0.65
12	24.8	29.0	28.1	28.5	8.4	11	27.9	0.6	0.43	0.58
13	25.6	29.7	26.4	26.8	8.6	14	27.9	0.5	0.07	0.94
14	24.5	29.2	25.2	25.8	7.8	20	27.9	1.6	0.19	0.81
15	22.3	28.7	25.6	25.9	7.8	29	28.0	2.9	0.53	0.47
16	24.2	28.8	22.8	23.1	9.4	14	28.0	0.5	0.09	0.91
17	22.0	28.2	25.3	25.6	7.6	22	28.0	3.1	0.85	0.15
18	23.6	29.6	23.3	24.2	9.2	17	28.0	1.0	0.51	0.49
19	25.0	29.3	24.6	25.0	8.8	19	28.0	0.7	0.53	0.47
20	24.6	29.8	22.6	22.7	9.0	15	28.0	0.6	0.92	0.08
21	22.0	28.3	21.3	21.4	7.7	21	28.0	3.5	0.83	0.17
22	23.6	28.3	21.2	21.2	8.0	19	28.1	1.7	0.96	0.04
23	20.6	28.8	17.9	18.0	8.0	12	28.1	2.6	0.97	0.03
24	24.5	29.4	18.3	18.9	9.0	34	28.1	0.7	0.97	0.03

### Spawning frequency analysis

An analysis of seasonal spatfall frequency of oysters in the areas flanking the contact zone was conducted, to determine whether the seasonal timing of spawning was similar between northern and southern populations. Spatfall numbers were compiled from long-term fishery independent monitoring data acquired by Texas Parks and Wildlife. Spat were extracted from reef material using a bottom dredge. Dredges were 495 mm wide and 241 mm high and were pulled at 3 mph for 30 s over the contour of known reef habitat. Spat were defined as settlement of oysters ranging in width from 5 to 25 mm. Spat counts in the Laguna Madre were made from 720 dredge pulls made on random reefs between the years 1986 and 1991. Spat counts in San Antonio Bay, Matagorda Bay, and Galveston Bay were made from 27,293 dredge pulls on random reefs made between the years 1984 and 2012. These areas occur outside of the contact zone, and are representative of areas in the southern and northern populations, respectively (see Results). Data were aggregated seasonally, with seasons defined as winter (Dec – Feb), spring (Mar – May), summer (Jun – Aug) and fall (Sept – Nov). The spawning frequency in each season was estimated separately for each area, and was calculated as total spat presence in each season, divided by total spat presence overall.

## Results

In macrogeographic samples, there was evidence for deviation from Hardy–Weinberg expectations at 7/11 microsatellite markers, all in the direction of heterozygote deficiency (Table 2). Examination of sample-specific *F*_IS_ suggested that a majority of deviations from Hardy–Weinberg occurred in central-coast samples, which in most cases were shown to be mixed population samples (*Q* < 0.70, Table 1).

Macrogeographic samples generally fell into two groups during the course of the exploratory structure analysis. Samples located north of Aransas Bay generally comprised a single genetic cluster, with the mean individual *Q* > 0.8 of structure cluster one (Fig. 2). In contrast, samples south of Corpus Christi bay had mean individual *Q* > 0.8 of structure cluster two. Within the Aransas and Corpus Christi bay area where additional micro-geographic samples were taken, the genetic structure of oysters varied from reef to reef (Fig. 3). Seven samples in this area were determined to be significantly admixed, meaning that: (1) they had mean individual *Q* < 0.7 relative to both structure clusters, and (2) they were the minimal set of low-*Q* samples that could be removed from the AMOVA model to yield a non-significant *F*_*sc*_, meaning allele frequencies across samples within regions were not significantly different. When admixed samples were removed, the among-sample divergence in AMOVA was not significantly different from zero (*F*_*sc*_ = 0.005, *P* = 0.092). Of the remaining samples in the Aransas/Corpus Christi area, seven were categorized as northern (structure cluster 1, *Q* > 0.8) and 11 were categorized as southern (structure cluster 2 *Q* > 0.8).

The seven mixed samples indicated high levels of linkage disequilibrium when grouped, with 38/55 pairwise comparisons between loci resulting in significant LD after tablewide correction (compared with 1/55 significant comparisons in the northern group and 6/55 significant comparisons in the southern group). These samples also deviated significantly from Hardy–Weinberg proportions at 9/11 loci, with heterozygote deficits in all cases. The AMOVA model between northern and southern regions (with admixed samples removed) indicated significant genetic divergence (*F*_*ct*_ = 0.392, *P* < 0.001).

**Figure 4:**
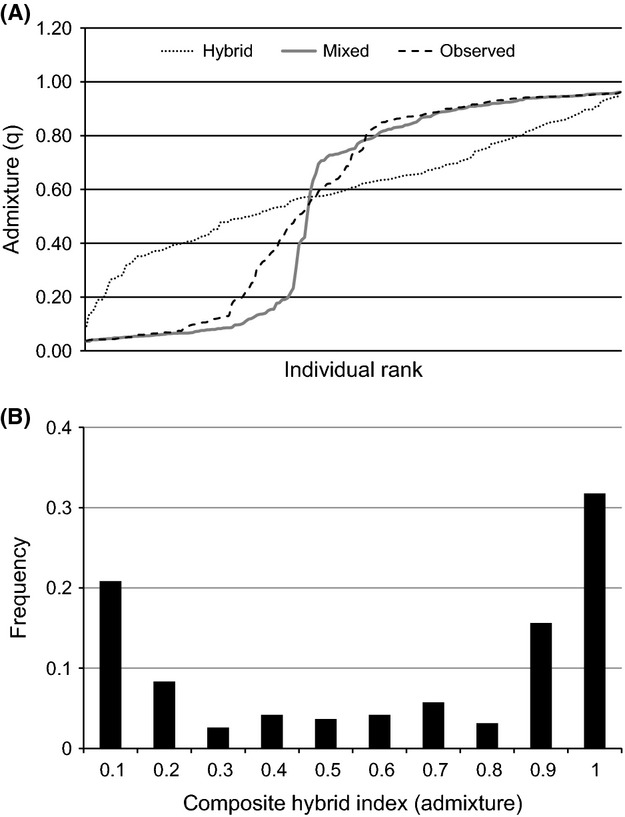
The distribution of admixture scores for eastern oysters in the transition zone between northern and southern populations of eastern oysters. In (A), the dashed line is the observed distribution, while the solid gray and dotted black lines are simulated distributions under the assumption of mechanical mixing, and hybrid swarm, respectively. In (B), the hybrid index plot is the distribution of hybrid index scores (based on individual admixture coefficients) across all individuals.

There was no significant difference in the distribution of admixture scores among individuals on the seven mixed reefs (Kruskal–Wallis *χ*^2^ = 7.75, df = 6, *P* = 0.257). Analyzing these seven samples together, the mechanical mixing model was a better fit of the observed distribution of admixture scores than was the hybridization model (Fig. 4A). The Kolmogorov–Smirnov test indicated that the mechanical mixing model was a reasonable fit for the data (*K*_sa_ = 0.87, *P* = 0.439), and the hybrid model was rejected as the underlying distribution (*K*_sa_ = 3.22, *P* < 0.001). This result was corroborated by the hybrid index plot, which was strongly bimodal with nearly pure parental forms outnumbering highly admixed individuals (Fig. 4B).

**Figure 5:**
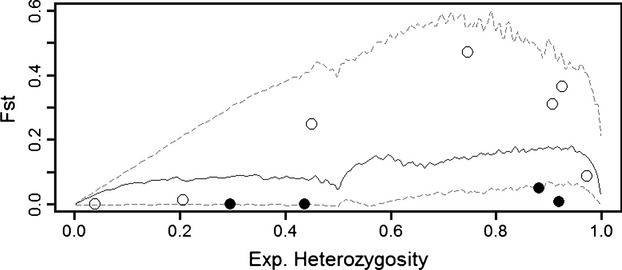
Plot of the distribution of heterozygosity and *F*_ST_ of microsatellite markers used to examine eastern oysters. The median *F*_ST_ (solid line), upper, and lower 95% confidence intervals (dashed lines) were generated via simulations using the method of Beaumont and Nichols (1996) as implemented in the software fdist2. Bolded circles are outlier loci, all other loci are empty circles.

**Figure 6:**
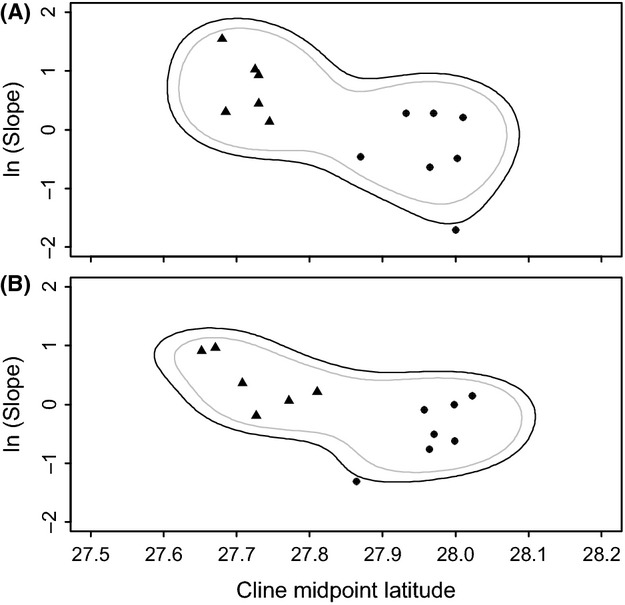
Co-Co plot of cline coincidence (midpoint) and concordance (slope), generated from 13 clinal loci observed in the current study (circles) and a previous study of eastern oyster population genetics (King et al. 1994; triangles). The density curves around the data are the 95% (gray) and 99% (black) confidence intervals of bivariate kernel volume calculated in the R package ks. Two plots were generated, including (A) one with data obtained using all samples from both the current study and King et al. (1994), and (B) only samples which were identical in their location in both studies.

The genomic outlier test (fdist2 analysis) suggested that none of the microsatellite markers were high *F*_ST_ outliers (no directional selection) but three were marginally low *F*_ST_ outliers (*P* ∼ 0.02, Rucv52, Cvi2j24, Cvi1j14) and Cvi9 was more extreme (*P* < 0.0001, indicating balancing selection, Fig. 5). Of the microsatellites analyzed here, 7/11 had clinal macrogeographic allele frequency variation, including one locus that was identified as a balancing selection outlier. An additional 6/15 allozymes from King et al. (1994) showed a clinal pattern and were included in the cline analysis. There were significant differences between microsatellite and allozyme data sets in both cline slope (*t* = 2.67, *P* = 0.0367) and mid-point (*t* = −11.44, *P* < 0.001). Both 95% and 99% density curves constructed around midpoint/slope plot points suggested a single outlier across all data, which was the microsatellite Cvi9 (Fig. 6A). The slope of the cline at this locus was significantly low. A second cline analysis using only the sample locations identical between the current and previous study (King et al. 1994) yielded a similar result of a single outlier, Cvi9 (Fig. 6B), as well as significant differences between cline slope (*t* = 3.11, *P* = 0.010) and midpoint (*t* = −7.83, *P* < 0.001).

None of the hydrological variables used in GESTE were significantly correlated with sample-specific genetic structure. In the macrogeographic analysis, the model which included only the model constant had the highest posterior probability (*P* = 0.627), as opposed to the next most likely model which included salinity but had a low posterior probability (*P* = 0.056). In the microgeographic analysis, the model which included only the constant again had the highest posterior probability (*P* = 0.144), as opposed to the next best model which included overall salinity and summer salinity (*P* = 0.113).

**Figure 7:**
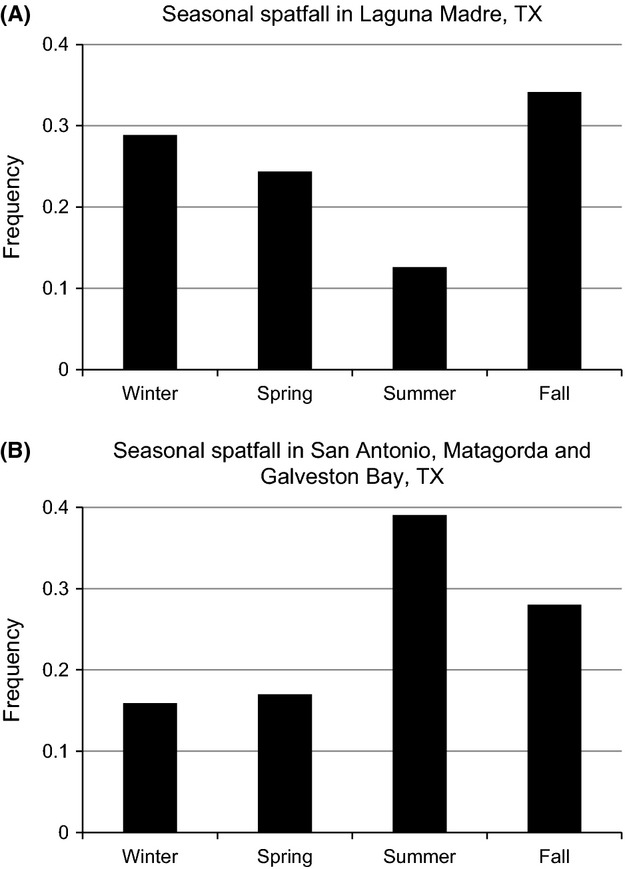
Seasonal spatfall frequency of eastern oysters based on long-term monitoring data from Texas Parks and Wildlife. Seasons are broken down as winter (December–February), spring (March–May), summer (June–August) and fall (September–November). Spat were defined as settlement of oysters ranging in width from 5 to 25 mm and were extracted from reef material using a bottom dredge. The *y*-axis represents the total spat presence in each season, divided by total spat presence overall (bars). Dredges were 495 mm wide and 241 mm high and were pulled at 3 mph for 30 s over the contour of known reef habitat in each area. Spat counts in the Laguna Madre (A) were made from 720 dredge pulls made on random reefs between the years 1986 and 1991. Spat counts in San Antonio Bay, Matagorda Bay, and Galveston Bay (B) were made from 27,293 dredge pulls on random reefs made between the years 1984 and 2012. These areas occur outside of the area of overlap between oyster populations and represent spawning frequency in the southern and northern populations, respectively.

Dredge monitoring data indicated a potential difference in spawning times between northern and southern populations (Fig. 7). Spawning in San Antonio, Matagorda, and Galveston bays (all north of the hybrid zone) occurred throughout the year, but peaked in summer. In contrast, spawning in the Laguna Madre (south of the hybrid zone) occurred throughout the year but was infrequent in summer.

## Discussion

The data examined in this study are consistent with earlier studies which suggest two distinctive populations of eastern oysters occurring in the western Gulf of Mexico (Groue and Lester 1982; Buroker 1983; King et al. 1994; Hoover and Gaffney 2005; Varney et al. 2009). Southern Aransas Bay and Corpus Christi Bay represent a transition zone between these divergent populations. A mixture of northern and southern individuals on sampled reefs occurred frequently in southern Aransas Bay and in Corpus Christi Bay, and the mixture proportion decreased rapidly with increasing distance from this transition zone. Prior to this study, King et al. (1994) conducted the most extensive population genetics study of oysters in Texas and concluded that gene flow was rare between northern and southern populations. Interpretation of these earlier data centered partly upon the finite and heterogeneous distribution of oyster reef habitat in the Laguna Madre. In particular, it was thought that geographic isolation and genetic drift may have played an important role in the observed allele frequency clines between populations (King et al. 1994). However, the current data demonstrate overlap between the populations, refuting the notion of geographic isolation. One plausible interpretation of the observed pattern in both the current study, and the King et al. (1994) study is that (1) one or both populations have recently expanded, and (2) oysters in Corpus Christi Bay and Aransas Bay represent a secondary contact zone between previously isolated populations. Interestingly, King et al. (1994) sampled the same reef in Corpus Christi Bay in 1989 that was sampled in the current study (Indian Point Reef). Although the earlier study found this reef to have allele frequencies at multiple loci that clustered significantly with the northern population, in the current study this reef was mixed, and more heavily influenced by southern population alleles. This result is supported by northerly shifts in the geographic midpoint of clinal genetic loci, by an average of 27 km over an approximately 23-year span (oysters collected in 1989 vs. 2012). This systematic shift in clinal midpoint among multiple independent loci suggests expansion of the southern population into an area previously inhabited primarily by northern oysters.

The physical and hydrological changes associated with the opening of the GIWW have occurred recently enough (the project was completed in 1949) that this represents a plausible mechanism behind recent secondary contact. The GIWW is the only consistent means of water exchange between the two basins of the Laguna Madre. Prior to the opening of this channel, salinities as high as 100 ‰ were commonly recorded in the upper Laguna Madre, and as high as 60 ‰ in the lower Laguna Madre (Quammen and Onuf 1993), resulting in numerous salinity-related fish kills (Simmons 1957; Gunter 1967). The opening of the GIWW not only allowed for tidal exchange between basins, but has also moderated salinity (Simmons 1957; Quammen and Onuf 1993) and has reduced the frequency and magnitude of salinity-related fish kills (Simmons 1957; Gunter 1967). The development of eastern oyster larvae from eggs generally requires salinities lower than 39 ‰ (Amemiya 1926; Clark 1935; although see Breuer 1962), and is optimum at around 27.5 ‰ (Davis 1958). These conditions were uncommon in both basins of the Laguna Madre prior to construction of the channel. The changes in hydrology subsequent to the opening of the channel have resulted in increased habitat quality for finfish (Simmons 1957) and seagrasses (Quammen and Onuf 1993) and have improved salinity conditions for supporting migration of planktonic eggs and larvae.

In addition to improving abiotic conditions for planktonic transport, the opening of the GIWW has also created the geophysical patterns (winds and tides) necessary for south-to-north transport of oyster larvae during peak spawning. Although oysters spawn year-round in Texas, spawning is at its peak in the spring, summer and early fall (Hopkins 1931; Breuer 1962; Fig. 7). Coincident with this peak in spawning there is both strong southerly offshore currents (Zavala-Hidalgo et al. 2003) as well as persistent southeast wind stress (Gutierrez de Velasco and Winant 1996) over the western Gulf of Mexico. As a result, wind-driven currents, which are the -primary means of larval transport in the shallow Laguna Madre, occur from south-to-north almost year-round and particularly during spring and summer (Simmons 1957; Breuer 1962). Thus a south-to-north system of larval transport from the southern end of Lower Laguna Madre into Upper Laguna Madre, and finally toward the southern end of Corpus Christi Bay, is expected based on qualitative interpretation of the hydrological data.

In the context of a secondary contact zone, it is interesting to reconsider the genome scan results and ask whether this small sample of markers mis-characterized the genomic average *F*_ST_ and led to an inference of balancing selection for four markers that simply experience high gene flow. In other words, if this population transition is similar to that documented for eastern oysters at Cape Canaveral, Florida (Murray and Hare 2006), then we might expect a minority of the genome to experience persistent barriers to gene flow (by indeterminate mechanisms). Comparison of population samples on either side of the Cape Canaveral cline showed only 1–3% of AFLP markers had significantly differentiated allele frequencies (Murray and Hare 2006). If the same is true in Texas, then it is possible for sampling error to misrepresent the average genomic *F*_ST_ among 11 independent loci. Whether or not the fraction of differentiated loci in Texas is 1, 3 or more percent, the alternative pattern of spatial homogeneity may simply reflect chromosomal regions that have escaped the population boundary constraints experienced by other loci. These could include loci under different forms of selection including balancing. However, we should also expect that some neutral loci did not become differentiated by drift during vicariance and therefore show no cline today. Such variance in genetic differentiation among loci can be expected in secondary contact zones (Latta 2003), due to the differential effects of indirect selection acting on distantly linked loci (Bierne et al. 2003) as well as differences in recombination across loci (Ford 2002). In any event, the high variance in divergence among loci underpins the points that (1) genomic sampling in this study is insufficient to disentangle the effects of sampling error versus selection acting against multiple genetic loci, and (2) genome scans can be misleading about signatures of selection unless genomic sampling is sufficiently large as to generate accurate estimates of neutral genomic variance (Bierne et al. 2003; Murray and Hare 2006).

In the case of the locus that showed evidence for balancing selection using both the fdist2 and cline analysis (Cvi9), it is possible that this result was an artifact as well. The highest estimate of *F*_IS_ among all loci (*F*_IS_ range 0.11–0.18) was found at Cvi9, indicating the potential for incorrect genotyping due to null alleles. This locus had an *F*_ST_ value that was within the range exhibited by other markers, and its outlier status using the fdist2 approach was primarily the result of the upward shift of the 95% confidence interval due to the high heterozygosity of this marker. Given this, and the previously discussed pitfalls of genome scans with limited genomic sampling, the finding of balancing selection at Cvi9 must be considered dubious.

The admixture data from individuals sampled directly in the eastern oyster hybrid zone demonstrate a bimodal hybrid pattern. Additionally, the deviation from HWE in these samples along with observed linkage disequilibrium suggest hybridization is occurring at a rate that is lower than what would be expected assuming random mating within the hybrid zone. These results may indicate the presence of reproductive isolation despite overlapping distributions. One potential explanation for this result is that differences in spawning times between northern and southern oyster populations may limit opportunities for hybridization (pre-mating isolation). Initiation of spawning in eastern oysters is cued mainly by water temperature (Stauber 1950; Barber et al. 1991), and adaptive genetic differences in spawning temperatures among localized races of oysters have been previously demonstrated (Barber et al. 1991). The genetic divergence between populations is substantial (*F*_*ct*_ = 0.392), and oysters from South Bay, Lower Laguna Madre have previously been suggested to be a distinct physiological race based upon the finding that individuals in this population spawned and achieved rapid growth in salinities >40‰ (Breuer 1962). There is also evidence for subtle differences in spawning times between northern and southern populations based upon spat formation. Texas Parks and Wildlife long-term monitoring data suggest that spawning in the Laguna Madre occurs year-round but is less frequent in summer (June–Aug), whereas spawning peaks during summer in bays north of the transition zone (Fig. 7). This difference is reflected in the literature as well; peak spatfall in spring and fall was noted by Breuer (1962) in the Laguna Madre, in contrast to primarily summer spatfall in Galveston bay noted by Hopkins (1931). Given previous findings of genetic adaptation to spawning temperatures (Barber et al. 1991), the potential for differences in spawning cues between northern and southern oysters in Texas is high and represents a testable hypothesis in explaining low gene flow between northern and southern populations.

These genetic data interpreted in the context of recent hydrologic alterations related to the GIWW suggest that oysters in the Corpus/Aransas area represent divergent populations, which have come into secondary contact, and further that the distribution of these populations may continue to be dynamic. There are two potential pitfalls to the interpretation of a moving hybrid zone to explain the difference in the current study and the previous (King et al. 1994) study. First, only a single sample was taken in the hybrid zone in the earlier King et al. (1994) study. The present study indicates dramatic variation among reefs at small spatial scales in the hybrid zone, suggesting the potential for a mosaic hybrid zone whereby the distribution of each population is variable, and tied to environmentally patchy habitats. Such a mechanism has been suggested in previous hybrid-zone work on marine mussels (genus *Mytilus*) existing in near-shore areas in Europe (Bierne et al. 2002). The current study was not able to capture correlation between population identity and measured environmental variables, nor were there any qualitative observations of differences between reefs in the hybrid zone. However, the interpretation of a directionally moving hybrid zone versus a mosaic zone given our data set is equivocal.

Second, whereas King et al. (1994) based their assessment on protein-based allozyme markers, the current study uses DNA-based microsatellites, introducing a sampling bias that could influence the interpretation of a moving hybrid zone. Such a bias has been observed in *Crassostrea virginica* when comparing patterns of variation in allozymes (Buroker 1983) versus mtDNA (Reeb and Avise 1990) and single-copy nuclear DNA loci (Karl and Avise 1992) across the North American range of the species. However, in those studies, the difference was in the magnitude of divergence among loci at sampled areas, rather than in the location of shifts in clinal loci.

One expectation of a moving hybrid zone is that future samples taken in central Texas may yield a different distribution of each lineage than what has been reported here. In this light, future sampling efforts should attempt to duplicate the geographic coverage obtained in this study, to clarify the processes at work in driving the distribution of each population in the hybrid zone. The question of hybrid-zone movement is an important one in regard to management, because efforts attempting to maintain genetic purity of parental forms (avoidance of introgression) could actually favor an invading population (Buggs 2007). Additionally, it is not known whether southern population oysters will respond favorably to commercial harvest; while the Corpus Christi area has not been a centerpoint for the commercial oyster industry in Texas due to sparsely distributed reefs, commercial harvest in Aransas Bay, San Antonio Bay, Matagroda Bay, and Galveston Bay (all of which contain oysters from the northern population) is intensive. The implications of two divergent populations overlapping in the Corpus/Aransas Bay system, and the potential for long-term changes in the distribution of each population is difficult to predict. Sustainable harvest of oysters in the western Gulf of Mexico will thus rely on a more thorough understanding and monitoring of the dynamics of this system.

## Data Accessibility

Microsatellite genotype data for all samples: Gulf of Mexico Research Initiative Information and Data Cooperative, Harte Research Institute, https://data.gulfresearchinitiative.org/data/00.x000.000:0175/
